# Key Molecular Events in PM_2.5_-Induced Lung Injury: Autophagy and Ferroptosis Mediated by the *miR-212-5p*/*RASSF1* Axis

**DOI:** 10.3390/cells15090823

**Published:** 2026-04-30

**Authors:** Cuizhu Zhao, Yunna Jia, Xiqing Zhang, Zhenhua Ma, Xiaohui Du, Xiaojun Liang, Xiuzhen Yu, Yunhang Gao

**Affiliations:** 1 College of Animal Science and Technology, Jilin Agricultural University, Changchun 130118, China; zhaocuizhu2024@163.com (C.Z.); 15590074644@163.com (Y.J.); mazhenhua1030@163.com (Z.M.); duxiaohui2001@163.com (X.D.); 2 Animal Husbandry and Veterinary Research Institute, Jilin Academy of Agricultural Sciences, Changchun 130033, China; zhangxiqing1020@163.com; 3 Institute of Animal Science, Ningxia Academy of Agriculture and Forestry, Yinchuan 750002, China; lxj0520@163.com; 4 Agricultural Equipment Research Institute of Xinjiang Uygur Autonomous Region, Academy of Agricultural Sciences, Urumqi 830091, China

**Keywords:** PM_2.5_, *miR-212-5p*, *RASSF1*, lung injury, autophagy, ferroptosis

## Abstract

**Highlights:**

**What are the main findings?**

- *miR-212-5p* promotes PM_2.5_-triggered autophagy and ferroptosis.
- *RASSF1* alleviates PM_2.5_-induced autophagy and ferroptosis in RLE-6TN cells through the PI3K/AKT/mTOR pathway.

**What are the implications of the main findings?**

- *miR-212-5p* may be a critical mediator in PM_2.5_-induced alveolar epithelial cell injury.
- *RASSF1* and the PI3K/AKT/mTOR axis represent potential targets for preventing or treating PM_2.5_-related lung damage.

**Abstract:**

Fine particulate matter (PM_2.5_) can directly impact pulmonary epithelial cells, resulting in lung injury. While it is known that PM_2.5_ can alter the expression profile of microRNAs in the lung, its specific role in damaging pulmonary epithelial cells remains unclear. This study, therefore, employed RT-qPCR, Western blotting, and dual luciferase reporter assays to investigate the regulatory role of microRNAs in PM_2.5_-induced cellular damage. PM_2.5_ exposure induces oxidative stress, autophagy, and ferroptosis in rat lung alveolar epithelial cells (RLE-6TN). Further functional rescue experiments confirm that the ferroptosis-specific inhibitor Fer-1 can block PM_2.5_-induced ferroptosis. Bioinformatics analysis and validation indicate that *miR-212-5p* plays a crucial role by negatively regulating *RASSF1* through targeted inhibition. Overexpression of *miR-212-5p* activates the PI3K/AKT signaling pathway, thereby promoting autophagy and ferroptosis. However, when the expression of both *miR-212-5p* and *RASSF1* is suppressed, PM_2.5_-induced autophagy and ferroptosis are significantly alleviated by inhibiting the PI3K/AKT/mTOR signaling pathway. Rescue validation experiments demonstrated that, under PM_2.5_ exposure combined with *RASSF1* overexpression, *miR-212-5p* exacerbates the aforementioned cellular damage process. This study reveals that *miR-212-5p* regulates autophagy and ferroptosis by targeting *RASSF1*. These findings provide a multi-target intervention strategy for PM_2.5_-related lung diseases.

## 1. Introduction

Ambient fine particulate matter (PM_2.5_) refers to air pollutants with an aerodynamic diameter of ≤2.5 µm. Due to their tiny size and large specific surface area, these particles readily accumulate various toxic and harmful substances, can penetrate the airway’s defensive barriers, and settle in the alveolar region. Short-term or long-term exposure to PM_2.5_ is closely linked to the onset and progression of various respiratory diseases, such as chronic obstructive pulmonary disease (COPD) and asthma, and has become a major environmental risk factor contributing to the global burden of disease [1,2]. PM_2.5_ concentrations are typically higher within livestock and poultry housing in intensive farming systems than in outdoor environments [3]. This poses a serious threat not only to animal health, but also to the control of air quality in livestock and poultry housing. Furthermore, existing research indicates that it can damage lung cells by inducing autophagy, cell death and oxidative stress. Excessive exposure can also directly damage the alveoli. In contrast, low doses of PM_2.5_ induce cellular oxidative stress and activate autophagy, which clears damaged cellular components and organelles [4,5]. In recent years, ferroptosis has emerged as a promising therapeutic target for lung injury induced by PM_2.5_. High concentrations of PM_2.5_ trigger ferroptotic cell death by increasing intracellular reactive oxygen species (ROS) levels and suppressing the activity of superoxide dismutase, catalase, and glutathione peroxidase [6]. Alveolar epithelial cells are critical for protecting and regulating lung function, and damage to these cells caused by PM_2.5_ represents a core mechanism in the progression of lung injury. Understanding the autophagy and ferroptosis responses in alveolar epithelial cells exposed to PM_2.5_ is important for identifying new targets for intervention in lung injury research.

MiRNAs are endogenous, regulatory, non-coding, small RNA molecules that were first identified in *Caenorhabditis elegans*. Mutations in these molecules disrupt larval developmental timing. Although they do not encode proteins, they exert regulatory functions by binding to and downregulating the mRNA of the *lin-14* gene [7,8]. miRNAs participate in cellular biological processes by regulating the translation and degradation of target mRNA. Their role in lung injury has been demonstrated, such as miR-486 targeting *PTEN* and *FOXO* to exert lung-protective effects [9]. ZIF-8 nanocarriers use their ability to target macrophages to effectively deliver anti-inflammatory miRNAs and mitochondria, protecting hosts from lung injury [10]. Serum miRNAs also demonstrate potential as biomarkers for PM_2.5_ exposure [11]. Dysregulated miRNAs hold significant clinical potential as disease “molecular switches.” Although their involvement in regulating PM_2.5_-induced lung injury has been demonstrated, most related studies have been limited to single-cell death pathways, such as *miR-16-5p* regulating ferroptosis and *miR-33* regulating macrophage autophagy [12]. As the primary target organ of PM_2.5_ exposure, the damage and repair of alveolar epithelial cells directly determine the progression of lung injury. Previous studies have confirmed that *miR-212-5p* plays a crucial role in inducing lung injury; for example, *miR-212-5p* targets *ARAF* to regulate the MEK/ERK pathway, thereby modulating alveolar macrophage apoptosis [13] and targeting *Ptgs2* to prevent ferroptosis in *CCI* mice. Furthermore [14], *miR-212-5p* can also inhibit the *SIRT6-HIF-1α* signaling pathway to alleviate lung injury [15]. Our team initially used an independently established in vivo rat infection model, screened differentially expressed miRNAs through transcriptomic data analysis, and, through screening and bioinformatics analysis, identified *miR-212-5p*. It has been proven that its expression is upregulated under PM_2.5_ induction, negatively regulates *LAMC2* and *LAMA3*, and promotes alveolar macrophage apoptosis [16]. However, its role in regulating autophagy and ferroptosis remains unclear. The aim of this study is to elucidate the mechanism by which *miRNA-212-5p* regulates autophagy and ferroptosis in alveolar epithelial cells, as well as its role in lung injury pathogenesis.

*RASSF1* (Ras-related structural domain family 1) is a key regulator of cell death and the cell cycle. As a member of the Ras-related structural domain family, it is involved in various cellular functions, including signal transduction and cell cycle regulation. Abnormal expression of *RASSF1* is closely associated with excessive cell proliferation, dysregulated apoptosis, and various forms of cell death. And with an N-terminal Ras-association (RA) domain, a C-terminal coiled-coil domain, and a SARAH domain, the protein encoded by *RASSF1* can participate in interactions of key pathways such as Hippo and MAPK to regulate cellular oxidative damage [17]. As a key Hippo pathway regulator, the encoded *RASSF1A* protein indirectly modulates autophagy by activating *MST1*, which phosphorylates *Beclin1* at Thr108 (H3 domain) to block its interaction with *Vps34* and inhibit autophagosome formation; notably, *RASSF1* acts via upstream *MST1* activity rather than direct binding to core autophagy proteins [18]. Despite oxidative stress and lipid peroxidation driving ferroptosis, no direct studies have linked *RASSF1* to ferroptosis regulation. Although early studies suggest that *RASSF1* may play a role in cellular responses to oxidative stress [19,20], its role in ferroptosis and the mechanisms underlying its interactions with key proteins GPX4 and ACSL4 remain unclear. Therefore, elucidating the specific molecular mechanisms by which *RASSF1* contributes to PM_2.5_-induced lung injury is of great significance for gaining a deeper understanding of the pathogenesis of PM_2.5_-related lung injury and developing targeted interventions.

The PI3K/AKT pathway is a key intracellular signaling hub that regulates various cellular processes. When activated, PI3K generates second messengers (PIP2 and PIP3) by phosphorylating phosphatidylinositol, thereby activating AKT. AKT then regulates various cellular processes by phosphorylating downstream effector molecules. mTOR is a core intracellular regulatory protein that works with the PI3K/AKT pathway to coordinate cellular processes [21,22]. Research indicates that autophagy plays a crucial role in the PI3K/AKT/mTOR pathway. Following AKT activation of mTORC1, the resulting complex phosphorylates p62, promoting its binding to Keap1 and ultimately enhancing cellular antioxidant capacity and suppressing ferroptosis by degrading Keap1 [23]. Research indicates that cinnamaldehyde has a protective effect against cellular damage, modulating multiple cell death pathways via the PI3K/AKT signaling pathway [24]. Therefore, the PI3K/AKT/mTOR pathway could be targeted in the treatment of PM_2.5_-induced autophagy and ferroptosis.

This study aims to elucidate the key mechanisms by which PM_2.5_ induces autophagy and ferroptosis, focusing on the validated *miR-212-5p*/*RASSF1* axis identified through screening and dual luciferase assays. This axis is a critical regulator of the PI3K/AKT/mTOR pathway and exerts significant influence on the aforementioned processes. These findings provide theoretical support for the identification of potential therapeutic targets.

## 2. Materials and Methods

### 2.1. Preparation of PM_2.5_ in Cattle Barns

For this study, PM_2.5_ samples were collected from a cattle farm in northern China using a multi-flow particulate matter sampler. Previous studies have conducted a systematic analysis of the microbial composition and chemical composition of this batch of PM_2.5_. This batch of PM_2.5_ contains a rich diversity of bacterial communities, and its bioaerosol characteristics and potential health impacts have been described in detail. The chemical composition of this batch of PM_2.5_ has also been quantitatively analyzed in previous studies [25,26]. They were then eluted with ultrapure water, freeze-dried to produce a dry powder, and stored at −20 °C for future use [27].

### 2.2. Establishment of Cell Models

The RLE-6TN (rat alveolar epithelial cell line) and 293T (human renal epithelial cell line) were acquired from the Chinese Academy of Sciences’ Shanghai Cell Bank and utilized in the experiment. The cell culture system consists of high-glucose DMEM basal medium (Gibco BRL, Grand Island, NY, USA), supplemented with 10% FBS, 100 U/mL penicillin, and 0.1 mg/mL streptomycin (Beyotime Biotechnology, Shanghai, China). The cell culture was performed in a 37 °C incubator supplied with 5% CO_2_. The following experimental groups were established in this study: The control group (CK): cells cultured in a medium without PM_2.5_. The cells in the concentration gradient exposure group were exposed to PM_2.5_ at concentrations of 0 ug/mL, 60 ug/mL, 180 ug/mL, and 300 ug/mL for 24 h. To assess PM_2.5_ toxicity, cells were subjected to a concentration of 180 ug/mL over a time course (0, 12, 24, and 48 h). The following were used to elucidate the role of *miR-212-5p*: PM_2.5_ + miRNA mimics NC, PM_2.5_ + *miR-212-5p* mimics, PM_2.5_ + miRNA inhibitor NC, and PM_2.5_ + *miR-212-5p* inhibitor. These were cultured in media containing PM_2.5_ and negative controls for the mimics or inhibitors. PM_2.5_ + pcDNA3.1 and PM_2.5_ + OE-*RASSF1* were cultured in media containing empty vector and *RASSF1* overexpression plasmid, respectively, to investigate the function of *RASSF1*. To verify whether PM_2.5_ exerts its effects by inducing ferroptosis and whether this process is mediated by *miR-212-5p* and *RASSF1*, we conducted rescue experiments. In these experiments, cells were pretreated with the ferroptosis-specific inhibitor Fer-1 (MedChemExpress, Monmouth Junction, NJ, USA) for 1 h, and changes in the expression of ferroptosis-related markers were subsequently detected to assess the extent to which Fer-1 rescued ferroptosis. The small RNA molecules used in this study were synthesized by GenePharma (Shanghai, China), including *miR-212-5p* mimics and their negative control, and *miR-212-5p* inhibitors and their negative control. The corresponding sequences are detailed in Supplementary Table S1.

### 2.3. Cell Transfection

Once the cells reached 60–70% confluence, transfection of *miR-212-5p* mimics, inhibitors, and their respective negative controls (NCs) into RLE-6TN cells was performed with a dedicated miRNA reagent (Polyplus-Transfection, Illkirch, France). Transfection efficiency was then assessed by RT-qPCR.

### 2.4. Cell Counting Kit-8 Assay

This study used the CCK-8 assay kit (Beyotime Biotechnology, Shanghai, China) to assess the toxic effects of PM_2.5_ on RLE-6TN cells by assessing cell viability. A cell density of 5 × 10^3^ cells/well was plated in a 96-well plate. After 24 h, the cells were treated with a medium containing 60, 180, or 300 ug/mL of PM_2.5_ for a further 24 h. Subsequent measurement of absorbance at 450 nm (microplate spectrophotometer) (Thermo Fisher Scientific, Waltham, MA, USA) provided the data from which relative cell survival rates were derived.

### 2.5. Oxidative Stress Index and Fe^2+^ Level

Intracellular malondialdehyde (MDA) levels were quantified with the corresponding assay kit (Nanjing Jiancheng Biotechnology Research Institute, Nanjing, China). Reactive oxygen species (ROS) (cat. no. S0033S), intracellular superoxide dismutase (SOD) (cat. no. S0101S), and Fe^2+^ (cat. no. S1066S) were obtained from Beyotime Biotechnology (Shanghai, China). MDA content and SOD activity are expressed in nmol/mg protein and units (where one unit inhibits 50% of the reaction), respectively. Fe^2+^ levels are also expressed in nmol/mg of protein.

### 2.6. Real-Time Quantitative Polymerase Chain Reaction (RT-qPCR)

Total RNA was extracted using the UNiQ-10 Column Trizol Total RNA Isolation Kit (Sangon Biotech, Shanghai, China). Reverse transcription of mRNA was conducted with the PrimeScript™ RT Reagent Kit (Takara, Japan), which incorporates a gDNA Eraser step. Subsequent quantitative PCR (qPCR) amplification was conducted employing the TB Green^®^ Premix Ex Taq™ II kit (Takara, Japan). Reverse transcription of miRNA was carried out using the miRNA First Strand cDNA Synthesis Kit (Vazyme Biotech Co., Ltd., Nanjing, China). miRNA expression levels were then quantified via RT-qPCR with a miRNA Universal SYBR qPCR Master Mix (Vazyme Biotech Co., Ltd., Nanjing, China). *U6* and *GAPDH* were used as internal controls for microRNA (miRNA) and messenger RNA (mRNA), respectively. Quantitative real-time polymerase chain reaction (qPCR) was performed using a qTOWER^3^ G instrument (Analytik Jena AG, Jena, Germany). The 2^−ΔΔCT^ method was applied to determine the relative expression levels of both mRNA and miRNA. Detailed sequences of the primers used are provided in Supplementary Table S2.

### 2.7. Target Gene Prediction

We used the TargetScan (v5.0) bioinformatics prediction website to predict downstream target genes capable of binding to miRNAs. After comprehensively evaluating the complementarity of the binding sites and the conservation scores, we identified the *RASSF1* gene to which *miR-212-5p* is capable of binding in its 3′ UTR.

### 2.8. Construction of Overexpression Vectors

The *RASSF1* overexpression plasmid was acquired from Wuhan Miaoling Biotechnology Co., Ltd. (Wuhan, China) as a glycerol stock. We performed plasmid DNA amplification and extraction using a Plasmid Prep Kit (Omega Bio-Tek, Norcross, GA, USA) and subsequently transfected this purified plasmid into RLE-6TN cells for subsequent experiments. We assessed the efficiency of *RASSF1* overexpression via Western blot and RT-qPCR.

### 2.9. Vector Construction and Dual Luciferase Reporter Assay

To experimentally confirm the targeting of *RASSF1* by *miR-212-5p*, this study created luciferase and green fluorescent protein reporter plasmids corresponding to each. The wild-type reporter plasmid was constructed using the ClonExpress II Cloning Kit (Vazyme Biotech Co., Ltd., Nanjing, China). Specific fragments from the 3′ UTR region of the *RASSF1* gene were cloned into the pmirGLO dual luciferase reporter vector and the mVenus-C1 green fluorescent protein vector to create the *RASSF1*-WT and *RASSF1*-mVenus-WT plasmids. We then used the Mut Express II Mutagenesis Kit (Vazyme Biotech Co., Ltd., Nanjing, China) to introduce mutations into the binding sites of the aforementioned plasmids, successfully creating the *RASSF1*-MUT and *RASSF1*-mVenus-MUT mutant plasmids. We then extracted plasmid DNA using a miniprep kit for endotoxin-free plasmid DNA (Tiangen Biotech (Beijing) Co., Ltd., Beijing, China). The sequences of the primers used can be found in Supplementary Table S3.

### 2.10. Western Blotting

Total protein was extracted by lysing cells on ice in RIPA buffer (Beyotime, Shanghai, China) containing 1% PMSF and 2% protease/phosphatase inhibitors. Protein concentration was determined via the Enhanced BCA Kit (Beyotime). Proteins were separated by SDS-PAGE, transferred to a 0.45 µm PVDF membrane (Merck, KGaA, Darmstadt, Germany), and blocked for 15 min (Servicebio, Wuhan, China). Membranes were incubated with primary antibodies at 4 °C overnight, followed by secondary antibody (1:1000, Proteintech, Group, Inc., Wuhan, China) for 2 h, and three 10 min TBST washes. Luminescence was detected using a BeyoECL kit (Beyotime, China) on an Amersham Imager 680 (GE Healthcare, Life Sciences, Marlborough, MA, USA), and the expression of protein bands was analyzed using ImageJ 1.53t (National Institutes of Health, Bethesda, MD, USA) Antibodies used in this study included: anti-*RASSF1*, anti-p62, anti-GPX4, anti-ACSL4, anti-p-PI3K, anti-p-mTOR (1:1000, Abways, Shanghai, China); anti-AKT (1:5000), anti-mTOR (1:2000), anti-PI3K (1:1000), anti-p-AKT (1:1000), anti-β-Tubulin (1:1000), anti-LC3II, and anti-LC3I (1:1000, Proteintech Group, Inc., Rosemont, IL, USA).

### 2.11. Statistical Analysis

The complete dataset underwent statistical processing and graphical visualization through GraphPad Prism 8.0.1 (GraphPad Software, Inc., San Diego, CA, USA). Data analysis entailed unpaired *t*-tests and one-way ANOVA for inter-group comparisons, and Tukey–Kramer post hoc tests. Values with *p* < 0.05 were defined as statistically significant.

## 3. Results

### 3.1. PM_2.5_ Induces Decreased Activity and Oxidative Stress in RLE-6TN Cells

**Figure 1:**
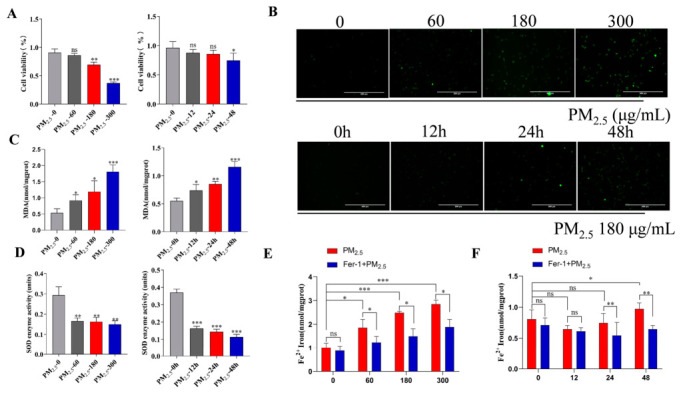
PM_2.5_ decreases the activity of RLE-6TN cells and induces cellular oxidative stress. (**A**) CCK-8 assay for cell viability. (**B**) ROS levels in PM_2.5_-exposed RLE-6TN cells (scale bar = 300 µm). (**C**) Changes in intracellular malondialdehyde (MDA) levels following PM_2.5_ stimulation of RLE-6TN cells. (**D**) The effects of PM_2.5_ stimulation on cellular superoxide dismutase (SOD) activity. (**E**) Changes in intracellular Fe^2+^ levels following treatment with different concentrations of PM_2.5_ alone or combined with Fer-1 pretreatment. (**F**) Changes in intracellular Fe^2+^ levels after PM_2.5_ intervention for different time points with or without Fer-1 pretreatment. Data are mean ± SD. Significance: ns (*p* > 0.05), * *p* < 0.05, ** *p* < 0.01, *** *p* < 0.001 vs. control.

RLE-6TN cells were exposed to PM_2.5_ at concentrations of 0, 60, 180, and 300 ug/mL for 24 h, and to 180 ug/mL PM_2.5_ for 0, 12, 24, and 48 h. CCK-8 assays showed that PM_2.5_ inhibited cell viability in both a concentration- and time-dependent manner (Figure 1A), indicating that PM_2.5_ exposure significantly impairs RLE-6TN cell activity. Further, intracellular ROS levels were quantified to assess PM_2.5_-induced oxidative stress. ROS levels increased concentration-dependently in the concentration-gradient group and time-dependently in the time-gradient group (Figure 1B). Further analysis revealed that the MDA concentration trends in both the concentration and time gradient groups largely aligned with ROS levels (Figure 1C). This was attributed to the sustained intensification of ROS-mediated lipid peroxidation reactions, resulting in the continuous accumulation of oxidatively degraded membrane lipids. We then measured SOD activity. In the concentration gradient group, SOD activity was markedly lower. In contrast, the time gradient group demonstrated a gradual, time-dependent decrease (Figure 1D). Fe^2+^ levels increased as PM_2.5_ concentrations rose, but decreased significantly following treatment with Fer-1 (Figure 1E). Over time, Fe^2+^ levels did not continue to decline as the duration of exposure increased; however, Fe^2+^ levels in the Fer-1-treated group remained lower overall than those in the group treated with PM_2.5_ alone (Figure 1F).

### 3.2. PM_2.5_ Induces Autophagy and Ferroptosis in RLE-6TN Cells

**Figure 2:**
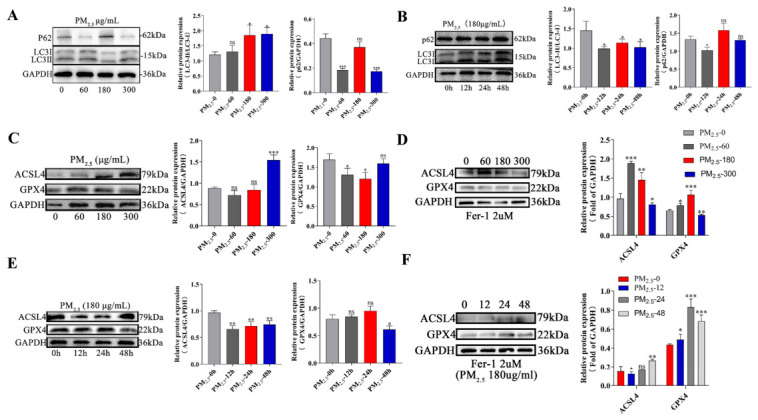
PM_2.5_ induces autophagy and ferroptosis in RLE-6TN cells. (**A**,**B**) Expression levels of LC3-II/LC3-I and p62 were detected by Western blotting. (**C**–**F**) Western blotting was used to detect the expression levels of the ferroptosis-related proteins ACSL4 and GPX4. Two groups were formed: one exposed to PM_2.5_ alone and one pretreated with ferritin (Fer-1) and then exposed to PM2.5. Data are presented as mean ± SD. Significance: ns = not significant (*p* > 0.05), *p* < 0.05 (*), *p* < 0.01 (**), *p* < 0.001 (***) vs. control group.

In order to investigate the effects of PM_2.5_ on autophagy and ferroptosis in RLE-6TN cells, we assessed the levels of both processes under PM_2.5_ exposure, using concentration (0 ug/mL, 60 ug/mL, 180 ug/mL, and 300 ug/mL) and time (0 h, 12 h, 24 h, and 48 h) gradients. Western blot results showed that the LC3II/LC3I ratio gradually increased in groups treated with different concentrations of PM_2.5_ as the concentration of PM_2.5_ rose. Meanwhile, p62 protein expression levels generally showed a downward trend (Figure 2A). Across the time gradient of PM_2.5_ exposure, compared with the 0 h control group, the LC3II/LC3I ratio was significantly reduced at 12 h of PM_2.5_ treatment, accompanied by downregulation of p62 protein expression. At 24 h of treatment, the LC3II/LC3I ratio was significantly higher than at 12 h, and p62 protein expression was also upregulated. By 48 h, the LC3II/LC3I ratio had decreased slightly, and p62 expression had also declined (Figure 2B). Western blot results showed that as the PM_2.5_ concentration increased, ACSL4 expression increased, while GPX4 expression decreased (Figure 2C). Compared to PM_2.5_ stimulation alone, pretreatment with ferritin (Fer-1) inhibited these changes (Figure 2D). Following PM_2.5_ treatment at different time points, ACSL4 was significantly downregulated after 12 h and remained at low expression levels after 24 and 48 h. GPX4 protein levels showed no significant changes after 12 or 24 h treatment, but were significantly downregulated after 48 h (Figure 2E). Furthermore, pretreatment with the ferroptosis-specific inhibitor Fer-1 significantly reduced the PM_2.5_-induced decrease in GPX4 protein levels (Figure 2F).

### 3.3. miR-212-5p Promotes Oxidative Stress, Autophagy, and Ferroptosis in Cells

**Figure 3:**
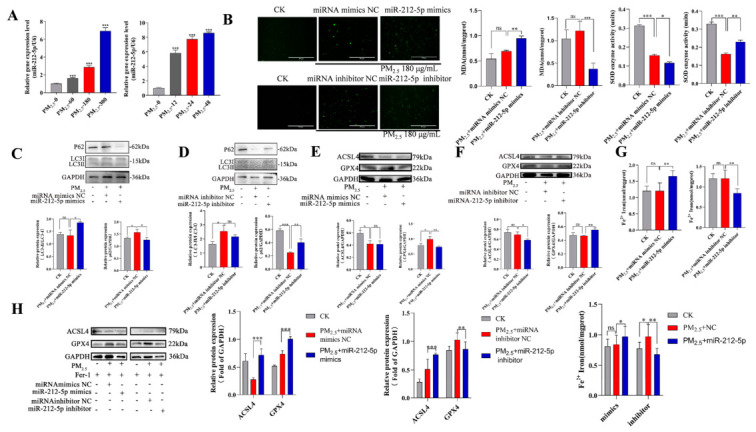
*miR-212-5p* promotes cellular oxidative stress, autophagy, and ferroptosis. (**A**) RT-qPCR of *miR-212-5p* in RLE-6TN cells treated with 180 ug/mL PM_2.5_. (**B**) Intracellular ROS, MDA, and SOD levels in PM_2.5_-treated RLE-6TN cells transfected with *miR-212-5p* mimics or inhibitor (scale bar = 300 µm). (**C**,**D**) Expression of the autophagy-related proteins LC3-II/LC3-I and p62 in RLE-6TN cells stimulated with 180 ug/mL PM_2.5_, as detected by Western blotting. (**E**,**F**) Expression of ferroptosis-related proteins ACSL4 and GPX4 in RLE-6TN cells stimulated with 180 ug/mL PM_2.5_ was detected by Western blotting. (**G**) Fe^2+^ levels were measured in cells from the *miR-212-5p* mimic and inhibitor groups, respectively. (**H**) The expression levels of ferroptosis-related proteins ACSL4 and GPX4, as well as Fe^2+^ content, were assessed by Western blotting following Fer-1 pretreatment. Data are mean ± SD. Significance: ns (*p* > 0.05), * *p* < 0.05, ** *p* < 0.01, *** *p* < 0.001 vs. control.

Exposure to PM_2.5_ alters the expression profile of miRNAs. This study revealed that the expression of *miR-212-5p* is upregulated under PM_2.5_ stimulation (Figure 3A). To determine the involvement of *miR-212-5p* in PM_2.5_-induced, oxidative stress-mediated autophagy and ferroptosis, we conducted validation experiments using both overexpression and knockdown approaches (i.e., *miR-212-5p* mimics and inhibitors). Elevated ROS and MDA levels indicate that *miR-212-5p* promotes oxidative stress by increasing the production of reactive oxygen species and malondialdehyde, while significantly reducing SOD activity. The ROS and MDA results indicate that *miR-212-5p* promotes the accumulation of cellular reactive oxygen species and malondialdehyde, while significantly reducing SOD activity (Figure 3B). This suggests that *miR-212-5p* enhances the body’s response to oxidative stress. Western blot analysis revealed that transfection with *miR-212-5p* mimics decreased p62 protein expression and increased the LC3II/LC3I ratio compared with the control group (Figure 3C). In contrast, transfection with the *miR-212-5p* inhibitor increased p62 protein expression and decreased the LC3II/LC3I ratio (Figure 3D). Further analysis of the expression of ferroptosis-related proteins revealed that GPX4 expression is downregulated by *miR-212-5p* mimics, but there is no significant effect on ACSL4 expression (Figure 3E). In contrast, the expression of GPX4 is upregulated, and the expression of ACSL4 is downregulated by the *miR-212-5p* inhibitor (Figure 3F). To further validate the effect of *miR-212-5p* on intracellular Fe^2+^ levels and the role of Fer-1, the results showed that *miR-212-5p* mimics significantly promoted Fe^2+^ accumulation (Figure 3G), pretreatment with Fer-1 significantly reduced the Fe^2+^ elevation induced by *miR-212-5p* mimics and reversed the regulatory effects of *miR-212-5p* mimics on ACSL4 and GPX4 protein expression (Figure 3H). The above experimental results demonstrate that *miR-212-5p* promotes PM_2.5_-induced cellular oxidative stress, autophagy, and ferroptosis.

### 3.4. miR-212-5p Regulates Autophagy and Ferroptosis Through Modulation of the PI3K/AKT/mTOR Pathway

**Figure 4:**
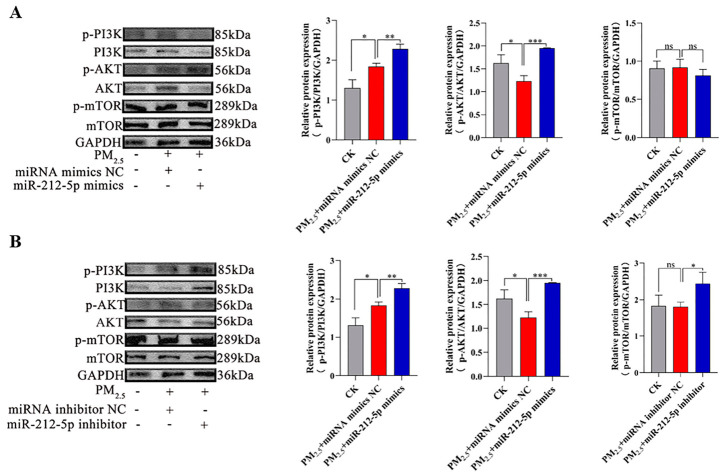
*miR-212-5p* regulates autophagy and ferroptosis via the PI3K/AKT/mTOR pathway. (**A**,**B**) Western blot analysis of p-PI3K/PI3K, p-AKT/AKT, and p-mTOR/mTOR in PM2.5-treated (180 ug/mL, 24 h) cells transfected with *miR-212-5p* mimics or inhibitor. Data are mean ± SD. Significance: ns (*p* > 0.05), * *p* < 0.05, ** *p* < 0.01, *** *p* < 0.001 vs. control.

Our preliminary work has verified the function of *miR-212-5p*. Further exploration of the mechanism of action of *miR-212-5p* is required. We analyzed its effects on the PI3K/AKT/mTOR signaling pathway by measuring the ratio of phospho-PI3K, phospho-AKT, and phospho-mTOR to their respective total protein levels. Western blot analysis showed that transfection with *miR-212-5p* mimics resulted in augmented activation of the PI3K/AKT axis, as evidenced by increased p-PI3K/PI3K and p-AKT/AKT ratios. Interestingly, there were no significant changes in p-mTOR/mTOR levels (Figure 4A). This suggests that, while *miR-212-5p* activates the PI3K/AKT signaling pathway, it has no significant effect on the mTOR pathway. Western blot analysis revealed that, in the *miR-212-5p* inhibitor group, the expression levels of p-PI3K/PI3K and p-mTOR/mTOR were elevated, whereas the expression of p-AKT/AKT was reduced (Figure 4B). Overall, these results suggest that *miR-212-5p* activates the PI3K/AKT signaling pathway.

### 3.5. miR-212-5p Directly Targets RASSF1

**Figure 5:**
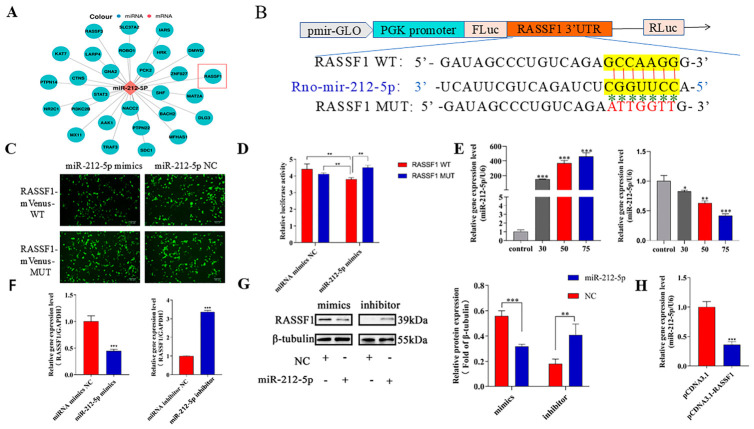
*miR-212-5p* directly targets *RASSF1*. (**A**) Predicted *miR-212-5p* targets (Targetscan v5.0). (**B**) Schematic: *RASSF1*-3′UTR & *miR-212-5p* reporter plasmid (red bars: binding sites; asterisks: mutation loci). (**C**) 293T co-transfection: *RASSF1*-mVenus-WT/MUT + NC/*miR-212-5p* mimics (scale bar = 300 µm). (**D**) Luciferase activity (293T: *RASSF1*-WT/MUT + *miR-212-5p* mimics/NC, 48 h post-transfection). (**E**) RT-qPCR: *miR-212-5p* mimic/inhibitor transfection efficiency. (**F**) RT-qPCR: *RASSF1* expression (*miR-212-5p* mimic/inhibitor-treated). (**G**) WB: *RASSF1* protein levels. (**H**) RT-qPCR: *miR-212-5p* expression following *RASSF1* overexpression. Data: mean ± SD. Significance: * *p* < 0.05, ** *p* < 0.01, *** *p* < 0.001 vs. control.

We have preliminarily demonstrated the role of *miR-212-5p*. To determine the detailed molecular mechanisms of cellular oxidative damage, bioinformatic prediction using TargetScan (v5.0) identified *RASSF1* as a putative target of *miR-212-5p* (Figure 5A,B). *RASSF1* (Ras-associated domain family member 1) is a key regulator of cell death and cell cycle processes. It is involved in important biological processes such as cell signaling and cell cycle regulation, and plays a crucial role in the cellular damage response. In addition, the *RASSF1* protein functions as a key node at the crosstalk of signaling pathways, thus arousing our research interest. To validate the interaction between *miR-212-5p* and *RASSF1*, we assessed their binding using a fluorescence microscope to observe the resulting fluorescence intensity. Co-transfection with the wild-type (WT) reporter plasmid showed that co-transfection with *miR-212-5p* decreased mVenus fluorescence intensity (Figure 5C). Subsequently, we transfected cells with WT and MUT plasmids containing the *RASSF1* 3′ translated region sequence. Dual luciferase assays revealed significantly reduced firefly luciferase activity in the WT plasmid group co-transfected with *miR-212-5p* (Figure 5D). The above data show that *RASSF1* and *miR-212-5p* can bind specifically. We subsequently validated the relationship between the two by transfecting RLE-6TN cells with *miR-212-5p* mimics and inhibitors to detect changes in *RASSF1* expression levels. First, the efficacy of the *miR-212-5p* mimics and inhibitors was assessed using RT-qPCR (Figure 5E). RT-qPCR and Western blot analysis indicate that *RASSF1* expression is downregulated by *miR-212-5p* mimics, whereas *RASSF1* expression is significantly increased by the *miR-212-5p* inhibitor (Figure 5F,G). The above fundings collectively demonstrate a negative correlation between *miR-212-5p* and *RASSF1*. Furthermore, we assessed the expression levels of *miR-212-5p* by transfecting cells with a *RASSF1* overexpression plasmid. This approach revealed downregulation of *miR-212-5p* level (Figure 5H), which provides additional validation of the negative correlation between the two.

### 3.6. RASSF1 Alleviates the Effects of PM_2.5_ on Cellular Oxidative Stress, Autophagy, and Ferroptosis by Regulating the PI3K/AKT/mTOR Pathway

**Figure 6:**
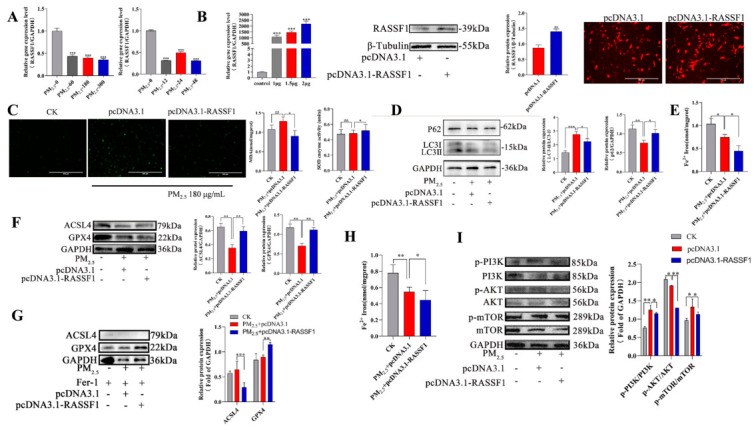
*RASSF1* reverses the effects of PM_2.5_ on cellular oxidative stress, autophagy, and ferroptosis via the PI3K/AKT/mTOR pathway. (**A**) *RASSF1* expression in PM_2.5_-stimulated cells. (**B**) Validation of *RASSF1* overexpression (RT-qPCR, WB, fluorescence; OE-*RASSF1*-mCherry; scale bar = 300 µm). (**C**–**I**) Assays in cells treated with 180 ug/mL PM_2.5_ (24 h) and transfected with OE-*RASSF1*: (**C**) Oxidative stress markers (ROS, MDA, SOD); (**D**) Autophagy-related proteins (LC3-II/LC3-I, p62); (**E**) Intracellular Fe^2+^ levels; (**F**) Ferroptosis-related proteins (ACSL4, GPX4); (**G**,**H**) Expression levels of the ferroptosis-related proteins ACSL4 and GPX4, as well as Fe^2+^ content, following Fer-1 pretreatment; (**I**) PI3K/AKT/mTOR pathway key proteins. Data: mean ± SD. Significance: ns (*p* > 0.05), * *p* < 0.05, ** *p* < 0.01, *** *p* < 0.001 vs. control.

Our attention has been drawn to *RASSF1*, a tumor suppressor gene whose encoded protein, *RASSF1A*, operates at the intersection of signaling pathway networks. To define the role of *RASSF1* in PM_2.5_-induced injury, we initially assessed its response to exposure. Both qPCR and Western blot analyses demonstrated a consistent downregulation of *RASSF1* following PM_2.5_ treatment. (Figure 6A). We subsequently constructed an *RASSF1* overexpression vector (OE-*RASSF1*-mCherry) and transfected it into RLE-6TN cells. The results of fluorescence analysis, qPCR, and Western blotting collectively demonstrated the successful establishment of an efficient *RASSF1* overexpression vector (Figure 6B). We transfected OE-*RASSF1*-mCherry into RLE-6TN cells in the presence of PM_2.5_. Detection of intracellular ROS, MDA, and SOD levels showed that *RASSF1* effectively alleviates PM_2.5_-induced oxidative stress in cells (Figure 6C). We subsequently detected changes in autophagy and ferroptosis via Western blotting. The results indicated downregulation of LC3II/LC3I and ACSL4 protein expression alongside upregulation of GPX4 and p62 protein expression, while Fe^2+^ content decreased. These results demonstrate that ferroptosis and autophagy within cells were suppressed (Figure 6D–F). Furthermore, Fer-1 pretreatment further enhances the regulatory role of *RASSF1* on ferrocytosis-related proteins, as evidenced by further downregulation of ACSL4 expression, further upregulation of GPX4 expression, and a significant reduction in intracellular Fe^2+^ levels (Figure 6G,H). Subsequent analysis of the PI3K/AKT/mTOR pathway revealed a downregulation of key phosphorylated proteins (p-PI3K, p-AKT, p-mTOR) along with their corresponding activation ratios following PM_2.5_ treatment. The above results suggest that activation of this signal is being suppressed (Figure 6I). Concurrently, this demonstrates that *RASSF1* alleviates cellular autophagy and ferroptosis by inhibiting the PI3K/AKT/mTOR signaling pathway.

### 3.7. The miR-212-5p/RASSF1 Axis Controls Autophagy and Ferroptosis by Affecting the PI3K/AKT/mTOR Signaling Pathway

**Figure 7:**
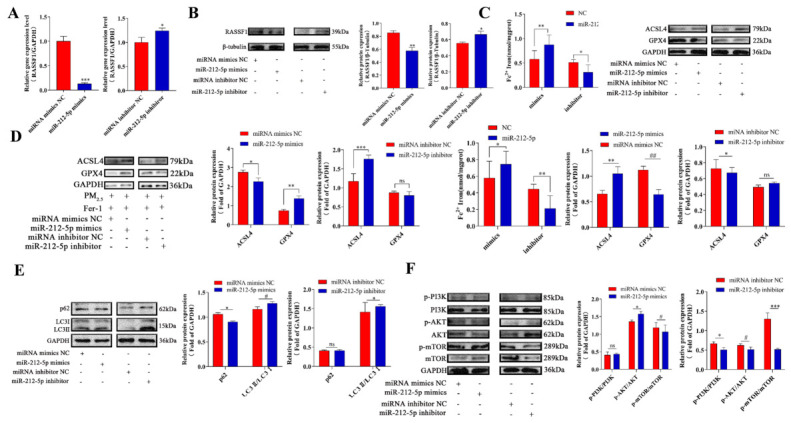
The *miR-212-5p*/*RASSF1* axis controls autophagy and ferroptosis in cells by regulating the PI3K/AKT/mTOR signaling pathway. (**A**,**B**). RT-qPCR (**A**) and Western blot (**B**) analyses were performed to investigate the expression of *RASSF1*, regulated by *miR-212-5p*, in *RASSF1*-overexpressing cells treated with 180 ug/mL PM_2.5_. (**C**) Shows the expression of ferroptosis-related proteins (ACSL4 and GPX4) in *RASSF1*-overexpressing cells 24 h after treatment with 180 μg/mL PM_2.5_, followed by transfection with *miR-212-5p* mimics or inhibitors. (**D**) Expression levels of ACSL4 and GPX4 proteins and Fe^2+^ content following Fer-1 pretreatment under the same conditions as in (**C**). (**E**) Western blot analysis of key proteins in the PI3K/AKT/mTOR pathway under the same conditions as in (**C**). (**F**) Expression levels of ferroptosis-related proteins ACSL4 and GPX4, as well as Fe^2+^ content, were detected by Western blot following Fer-1 rescue treatment. Data: mean ± SD. Significance: ns (*p* > 0.05), * *p* < 0.05, ** *p* < 0.01, *** *p* < 0.001 vs. control; # *p* < 0.05, ## *p* < 0.01 vs. control.

To ascertain whether the *miR-212-5p*/*RASSF1* axis is required within this pathway, we performed functional recovery experiments. Under conditions of PM_2.5_ exposure and *RASSF1* overexpression, we transfected cells with *miR-212-5p* mimics, *miR-212-5p* inhibitors, and corresponding negative controls (NCs). Changes in cellular autophagy, ferroptosis, and PI3K/AKT/mTOR signaling pathway were then assessed. qPCR and Western blotting results suggest that *RASSF1* expression is downregulated by *miR-212-5p* mimics and that *RASSF1* expression is further increased by the *miR-212-5p* inhibitor (Figure 7A,B). Functional recovery assays demonstrated that transfection with *miR-212-5p* mimics reversed the regulatory effects of *RASSF1* on the ferroptosis-related proteins ACSL4 and GPX4, resulting in upregulation of ACSL4 and downregulation of GPX4. In contrast, transfection with a *miR-212-5p* inhibitor led to further downregulation of ACSL4 and further upregulation of GPX4 (Figure 7C). Furthermore, Fer-1 pretreatment reversed the *miR-212-5p* mimic-induced upregulation of ACSL4, downregulation of GPX4, and accumulation of intracellular Fe^2+^ (Figure 7D). Further analysis of the expression of autophagy-related proteins revealed that transfection with *miR-212-5p* mimics resulted in decreased p62 protein expression and an increased LC3II/LC3I ratio; conversely, transfection with a *miR-212-5p* inhibitor led to further increases in p62 protein expression and a further decrease in the LC3II/LC3I ratio (Figure 7E). Signal transduction assays revealed that *miR-212-5p* mimics increased p-PI3K/PI3K and p-AKT/AKT levels, whereas the *miR-212-5p* inhibitor reduced the levels of these phosphorylations (Figure 7F). These results indicate that *miR-212-5p* targets *RASSF1* to regulate cellular autophagy and ferroptosis via the PI3K/AKT/mTOR signaling pathway.

## 4. Discussion

PM_2.5_ is a well-established pathogenic mediator of lung injury. This study uses concentration and time gradients to reveal that exposure to PM_2.5_ induces oxidative autophagy, oxidative stress, and ferroptosis in alveolar epithelial cells. Furthermore, we discovered that the *miR-212-5p*/*RASSF1* molecular axis is a key regulator governing this process. This axis promotes autophagy and ferroptosis by interfering with the PI3K/AKT/mTOR signaling pathway. These findings suggest that the *miR-212-5p*/*RASSF1* axis may be a potential target for interventions aimed at mitigating lung damage caused by PM_2.5_ in intensive livestock farming environments.

This study confirms that exposure to PM_2.5_ significantly induces the accumulation of reactive oxygen species, elevated malondialdehyde levels, and decreased superoxide dismutase activity in alveolar epithelial cells, indicating a typical state of oxidative stress. PM_2.5_ samples collected from intensive cattle barns exhibit unique physicochemical properties. Specifically, PM_2.5_ in cattle barns contains high levels of endotoxins and a diverse microbial community, which are unique components of biological aerosols and determine the toxic effects of PM_2.5_. The underlying molecular mechanism involves bioactive substances, such as endotoxins, that are carried by PM_2.5_. These substances can act as pathogen-associated molecular patterns and are efficiently recognized by pattern recognition receptors (e.g., TLR4) on the surface of alveolar macrophages. This directly activates the downstream NF-κB inflammatory signaling pathway and may also trigger *AMPK*-related metabolic stress pathways [28]. It is particularly important that this composition can amplify its toxic effects synergistically through multiple mechanisms. For example, endotoxins associated with PM_2.5_ trigger the TLR4/NF-κB pathway, which in turn activates the NLRP3 inflammasome to drive the maturation and release of proinflammatory cytokines, including IL-1β [29]. Research indicates that under specific exposure conditions, the *NLRP3/caspase-1* pathway can be activated in alveolar macrophages via an atypical inflammasome mechanism, thereby exacerbating lung injury [30]. Therefore, understanding the toxic effects of PM_2.5_ is important for assessing environmental risk factors in livestock farming. In this study, we focused on intact PM_2.5_ particles as the primary exposure target, with an emphasis on verifying the cellular damage and protective mechanisms they mediate. Endotoxin components such as LPS do indeed play a certain role, which is one of the limitations of this study. In future studies, we will further utilize PM_2.5_ reference standards and combine them with exogenously added LPS for intervention validation, in order to more accurately distinguish between the intrinsic toxicity of PM_2.5_ and the independent contributions of endotoxin and other components, thereby elucidating their specific damage pathways and molecular mechanisms in greater depth.

This study found that as PM_2.5_ concentrations increased, autophagy levels, ferroptosis markers, and intracellular iron levels were concurrently elevated. However, fluctuations in autophagy-related markers were observed over time within the 12–24 h window. This was characterized by the accumulation of p62 protein and a concurrent increase in the LC3-II/LC3-I ratio. These findings suggest that a specific step in the autophagy process may be restricted. Previous studies have demonstrated an association between the activation of autophagy and ferroptosis. It has been reported that *NCOA4*-mediated ferritin autophagy is a key pathway in PM_2.5_-induced iron-dependent toxicity [31,32]. This contrasts with the findings of this study, in which autophagy was initiated but ferroptosis-related markers were alleviated. This discrepancy may be due to the composition of PM_2.5_ particles. Specifically, exposure to PM_2.5_ may cause certain constituents, such as endotoxins and heavy metals, to disrupt lysosomal degradation within cells, thereby blocking the autophagic flux degradation pathway [33]. Conversely, impaired autophagy further impairs the transport of iron ions via autophagy-mediated ferritin, thereby indirectly alleviating iron overload and damage caused by lipid peroxidation, ultimately resulting in the mitigation of ferroptosis. Although the aforementioned experiments alleviated some aspects of ferroptosis, intracellular iron overload remains a key pathogenic factor that requires further elucidation in a clinical context. Excessive hydroxyl radicals can be generated by intracellular iron overload via the Fenton reaction. These radicals attack cell membrane lipids, leading to the accumulation of lipid peroxides and membrane rupture [34]. Clinically, iron-overloaded cells undergo ferroptosis, and increased alveolar permeability leads to pulmonary oedema and *hypoxemia* disrupting the alveolar–capillary barrier. Furthermore, iron overload leads to elevated levels of reactive oxygen species and enhanced oxidative stress at the cellular level. This activates inflammatory pathways such as NF-κB, which promotes the release of pro-inflammatory cytokines such as IL-6 and TNF-α by alveolar macrophages. This creates a vicious cycle of ‘iron overload–inflammation’, resulting in exacerbated pulmonary inflammation [35]. During the early stages of PM_2.5_ exposure, slight autophagy activation exerts a protective effect by clearing damaged organelles. However, prolonged exposure leads to fluctuations in related processes. Whether this phenomenon is caused by impaired autophagic flux or suppressed substrate degradation requires further verification using lysosomal inhibitors and transmission electron microscopy.

The results of this study show that *RASSF1* expression levels decreased significantly after 12 h of PM_2.5_ exposure but recovered somewhat by 48 h. We speculate that its expression and function are subject to complex regulation. As an important tumor suppressor gene, the protein encoded by *RASSF1* primarily participates in processes such as cell cycle regulation and the mediation of cellular damage within cells. On the one hand, *RASSF1*′s methylation function leads to its epigenetic silencing. For instance, *HOXB3* promotes *DNMT3B* upregulation, leading to its recruitment to the *RASSF1A* promoter and subsequent gene silencing via promoter DNA hypermethylation [36]. Conversely, PM_2.5_ originating from cattle barns contains microorganisms, endotoxins, and pathogens. Under specific stress conditions such as pathogen infection, *RASSF1* expression is upregulated to execute its biological functions. Research has revealed that *RASSF1* expression is significantly elevated in *TC-1* mouse lung epithelial cell lines infected with Pasteurella *multocida*. The substantial rise in *RASSF1* protein levels in lung tissues after Pasteurella *multocida* infection in mouse models suggests that this pathogen can activate the Hippo-Yap pathway by driving *RASSF1* expression, thereby executing related biological functions, such as apoptosis [37]. Therefore, stress-induced activation of inflammatory and stress-related signaling pathways, including NF-κB and RAS/ERK, occurs in cells after exposure to PM_2.5_. These pathways maintain *RASSF1* expression at a certain level. The interplay between these two mechanisms ultimately determines *RASSF1* expression levels during PM_2.5_ exposure, which in turn influences its role in cellular stress and death.

As a central integration hub for signal transduction, the PI3K/AKT/mTOR pathway mediates cross-talk between intracellular and extracellular signals, thereby regulating key cellular processes like cell survival, metabolism, autophagy, and death. This makes it a crucial player [38,39]. The study found that *miR-212-5p* activates the PI3K/AKT pathway but has no significant effect on the mTOR pathway. Under PM_2.5_ stimulation, the expression level of endogenous *miR-212-5p* in RLE-6TN cells may have reached a relatively high state, resulting in “saturation inhibition” of the downstream mTOR pathway. Therefore, additional overexpression of *miR-212-5p* did not further inhibit this pathway, resulting in no significant fluctuation in the p-mTOR/mTOR ratio; however, upon inhibition of endogenous *miR-212-5p* expression using an inhibitor, the suppression of its target genes was lifted, the mTOR pathway was activated, and significant changes were observed. In addition, *miR-212-5p* can target multiple genes simultaneously. Inhibiting *miR-212-5p* leads to the upregulation of multiple target genes, which may influence the mTOR pathway synergistically. For instance, research has demonstrated that *miR-212-5p* binds to genes such as *FAS* and *SCD1*, thereby regulating cellular lipid metabolism [40]. When its expression is inhibited, the repressive state of these downstream genes is lifted, thereby affecting pathway activity. Importantly, in a lung adenocarcinoma model, *miR-212-5p* was shown to exert its oncogenic effects by directly targeting and inhibiting Id3, thereby activating the PI3K/AKT signaling pathway [41]. We then validated *RASSF1*, which is a target gene of *miR-212-5p*. When it is overexpressed, the p-mTOR/mTOR ratio is altered (Figure 6I) and the activity of the PI3K/AKT signaling pathway is inhibited. However, mTOR signaling downstream of this pathway also shows a decreasing trend, though the regulatory effect is not particularly pronounced. This suggests that *Rassf1*’s intervention in the PI3K/AKT pathway may not be entirely mediated through the classical mTOR pathway, but rather through a more complex signaling network. Activating the mTOR pathway results in a statistically significant difference. Specifically, *RASSF1*, a multifunctional scaffold protein, stabilizes and activates the *FOXO* transcription factor. The latter acts as a negative regulatory node in the PI3K-AKT signaling pathway and further enhances the inhibitory effect on this pathway. This forms a refined negative feedback regulatory loop that effectively mitigates fluctuations in mTOR signaling [42]. This confirms not only that *RASSF1* is a functional target of *miR-212-5p*, but also corroborates the results from the inhibitor group. This suggests that the regulation of the PI3K/AKT/mTOR pathway by the *miR-212-5p*/*RASSF1* molecular axis is not simply linear, but is instead achieved through a regulatory network involving multiple key molecules and targets that work together synergistically. This discovery emphasizes the pivotal role of miRNAs in signaling transduction and the intricacy of their functions.

This study found that, under PM_2.5_ exposure, overexpression of *RASSF1* or inhibition of *miR-212-5p* reduced mTOR activity. Interestingly, however, autophagy and ferroptosis remained suppressed. This seemingly contradictory phenomenon was also confirmed by the study, which found that the Raf/MEK/ERK pathway is often highly activated in Ras-transformed cells and is not regulated by mTORC1. In these cells, inhibiting mTORC1 activity alone is insufficient to induce a complete autophagy process; the completion of autophagy depends not only on mTORC1 inhibition but may also be regulated by parallel signaling pathways such as Raf/MEK/ERK [43]. Therefore, the autophagy process may still be suppressed even when mTOR activity is inhibited due to regulation by other pathways. Research indicates that, in prostate cancers lacking PTEN (phosphatase and tensin homolog), AKT and mTOR signaling become decoupled, resulting in tumour cell resistance to AKT inhibitors. mTOR can become activated in the absence of AKT regulation. When exposed to PM_2.5_, cells experience oxidative stress and die, resulting in damage to organelles such as mitochondria. This, in turn, causes PTEN dysfunction. Dysfunction of PTEN leads to unregulated AKT-mediated mTOR activity, enabling the activation of the PI3K/AKT signaling pathway and suppression of mTOR activity by *miR-212-5p* mimics [44].

However, this study also has several limitations that point the way for future exploration. The co-activation of autophagy and ferroptosis suggests that they may be connected by a feedback loop, rather than existing in a simple parallel relationship. Ferroptosis, a type of programmed cell death characterized by lipid peroxidation accumulation, often requires autophagy to be involved. Studies have confirmed that the activation of autophagy does not occur in isolation but frequently coincides with ferroptosis. This is because autophagy activation triggers the upregulation of autophagy-associated genes, which in turn drives autophagosome biogenesis [45]. NCOA4, a receptor mediating the selective translocation of ferritin to lysosomes for degradation, is highly enriched in autophagosomes. This increases Fe^2+^ levels, thereby accelerating lipid oxidation. Consequently, autophagy activation is often accompanied by ferroptosis [46]. At the same time, PM_2.5_ can induce the upregulation of METTL3 (methyltransferase-like 3), which mediates the N6-methyladenosine (m6A) methylation of PINK1, thereby activating mitochondrial autophagy. Moderate activation eliminates damaged mitochondria and reduces ROS accumulation; however, excessive activation releases free iron, thereby exacerbating ferroptosis [47]. Therefore, autophagy and ferroptosis are closely interconnected. Although our findings validated each phenomenon independently, they failed to reveal the intricate interrelationships between them. Furthermore, the significance of this molecular axis in complex physiological environments needs to be confirmed through in vivo studies. Future work should focus on elucidating the interactions between this axis and other pathways, and on the functional differences between different *RASSF1* isoforms. This could reveal novel regulatory layers in the mechanisms underlying PM_2.5_-induced lung injury.

## 5. Conclusions

PM_2.5_ exposure upregulates *miR-212-5p* and downregulates its direct target *RASSF1* (negatively correlated), inducing oxidative stress, autophagy, and ferroptosis. *miR-212-5p* activates the PI3K/AKT pathway to promote autophagy and ferroptosis, whereas *RASSF1* alleviates them via inhibiting the PI3K/AKT/mTOR pathway. These findings clarify the regulatory mechanisms of *miR-212-5p* and *RASSF1* in these processes.

## Data Availability

The original contributions presented in this study are included in the article. Further inquiries can be directed to the corresponding author.

## Supplementary Materials

The following supporting information can be downloaded at: https://www.mdpi.com/article/10.3390/cells15090823/s1, Figure S1: *RASSF1* protein expression levels; Table S1. The sequence information of mimics and inhibitors; Table S2. The sequence information of RT-PCR and RT-qPCR; Table S3. The sequence information of PCR.
