# Supplementary material for: Key Molecular Events in PM2.5-Induced Lung Injury: Autophagy and Ferroptosis Mediated by the miR-212-5p/RASSF1 Axis
Source: Cells. 2026 Apr 30;15(9):823. doi: 10.3390/cells15090823 (PMC13162688; doi:10.3390/cells15090823)
Supplement: Supplementary file 1 [file cells-15-00823-s001.zip › cells-4266733-supplementary.pdf]

A

B

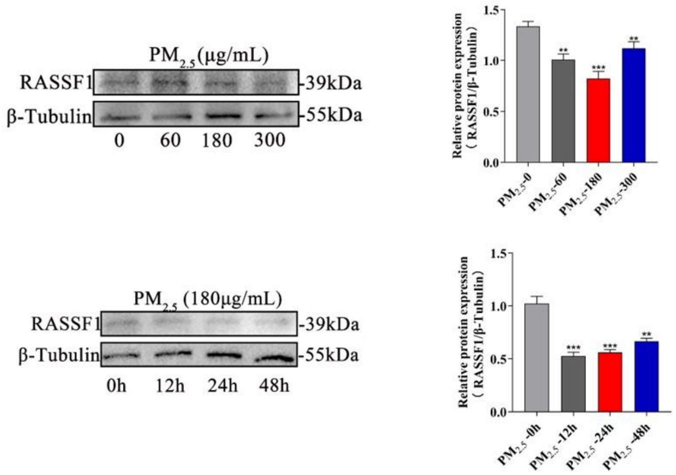


Figure S1：RASSF1 protein expression levels. （A） RASSF1 protein expression following $PM_{2.5}$ treatment at different concentrations (0, 60, 180, 300 μg/mL). （B）: RASSF1 protein expression following $PM_{2.5}$ treatment for different time periods (0, 12, 24, 48 h). Data are mean ± SD. Significance: ** $p < 0.01$, *** $p < 0.001$ vs. control.

| Primer ID | Sequences(5′ to 3′) |
|---|---|
| miR-212-5p mimics-sense | ACCUUGGCUCUAGACUGCUUACU |
| miR-212-5p mimics-antisense | UAAGCAGUCUAGAGCCAAGGUUU |
| miR-mimics NC-sense | UUCUCCGAACGUGUCACGUTT |
| miR-mimics NC-antisense | ACGUGACACGUUCGGAGAATT |
| miR-212-5p inhibitor | AGUAAGCAGUCUAGAGCCAAGGU |
| miR-inhibitor NC | CAGUACUUUUGUGUAGUACAA |

Table S1. The sequence information of mimics and inhibitors

| Primer ID | Sequences(5′ to 3′) | Tm (℃) | PCR program |
|---|---|---|---|
| qPCR-RASSF1-F | TGATCTTTCCCAAGCTGAGAC | 60 | 95℃ 30 sec, (95℃ 5 sec, Tm 60 sec) 40 cycles, 95℃ 5 sec, 60 ℃ 60 sec, 95 ℃ 1 sec, 37 ℃ 30 sec |
| qPCR-RASSF1-R | GGTGTAGGAGCCATCCTTATTC | 60 | |
| qPCR-GAPDH-F | CCTGCACCACCAACTGCTTA | 60 | |
| qPCR-GAPDH-R | CATCACGCCACAGCTTTCCA | 60 | |
| RT-miR-212-5p | GTCGTATCCAGTGCAGGGTCCGAGGTATTCGCACTGGATACGACAGTAAG | 74 | 25 ℃ 30 min |
| qPCR-miR-212-5p-F | CGCGACCTTGGCTCTAGACTG | 60 | 95℃ 5 min, (95℃ 10 sec, Tm 30 sec) 40 cycles, 95℃ 5 sec, 60 ℃ 60 sec, 95 ℃ 1 sec, 37 ℃ 30 sec |
| qPCR-miR-212-5p-R | AGTGCAGGGTCCGAGGTATT | 60 | |
| qPCR-U6-F | CTCGCTTCGGCAGCACA | 60 | |
| qPCR-U6-R | AACGCTTCACGAATTTGCGT | 60 | |

Table S2. The sequence information of RT-PCR and RT-qPCR

| Primer ID | Sequences(5′ to 3′) | Tm (℃) | PCR program |
|---|---|---|---|
| RASSF1-pmirGLO-WT-F | CCTTTATTTCCAGTCTTTCCTCAGGGT | 61 | 94℃ 3 min, (94 ℃ 30sec, Tm 30 sec, 72℃ 1 min) 30 cycles, 72 ℃ 10 min |
| RASSF1-pmirGLO-WT-R | GAGTCTAGGGCAACTTTCCCGTAGAG | 63 | |
| RASSF1-mVenus-WT-F | CGGGATCCGAGCTCGGTACGGCCAAGGTAGCTGCTGAGAA | 68 | |
| RASSF1-mVenus-WT-R | AGATCTCGAGATCTAGAGCTTCTCAGCAGCTACCTTGGCC | 68 | |
| RASSF1-pmirGLO-MUT-F | CCTTTATTTCCAGTCTTTCCTCAGGGT | 66 | 95℃ 30 sec, (95 ℃ 15 sec, Tm 15 sec, 72℃ 4 min) 30 cycles, 72℃ 10min |
| RASSF1-pmirGLO-MUT-R | GAGTCTAGGGCGTGTTTCCCGTAGAG | 66 | |
| RASSF1-mVenus-MUT-F | CGGGATCCGAGCTCGGTACAGAATTCGGATGTAGCTGCTG | 68 | |
| RASSF1-mVenusMUT-R | AGATCTCGAGATCTAGAGCAGCAGCTACATCCGAATTCT | 66 | |

Table S3. The sequence information of PCR
